# Phosphorylation of Neurofilament Light Chain in the VLO Is Correlated with Morphine-Induced Behavioral Sensitization in Rats

**DOI:** 10.3390/ijms24097709

**Published:** 2023-04-22

**Authors:** Yu-Xiang Zhang, Yuan-Mei Zhu, Xi-Xi Yang, Fei-Fei Gao, Jie Chen, Dong-Yu Yu, Jing-Qi Gao, Zhen-Nan Chen, Jing-Si Yang, Chun-Xia Yan, Fu-Quan Huo

**Affiliations:** 1 College of Forensic Medicine, Xi’an Jiaotong University Health Science Center, Xi’an 710061, China; 2 NHC Key Laboratory of Drug Addiction Medicine, Kunming Medical University, Kunming 650032, China; 3 The Key Laboratory of Environment and Genes Related to Diseases, Ministry of Education, Xi’an Jiaotong University, Xi’an 710061, China; 4 Department of Physiology and Pathophysiology, School of Basic Medical Sciences, Xi’an Jiaotong University Health Science Center, Xi’an 710061, China

**Keywords:** cAMP response element-binding protein (CREB), extracellular signal-regulated kinase (ERK), histone deacetylases, neurofilament light subunit (NF-L), ventrolateral orbital cortex

## Abstract

Neurofilament light chain (NF-L) plays critical roles in synapses that are relevant to neuropsychiatric diseases. Despite postmortem evidence that NF-L is decreased in opiate abusers, its role and underlying mechanisms remain largely unknown. We found that the microinjection of the histone deacetylase (HDAC) inhibitor Trichostatin A (TSA) into the ventrolateral orbital cortex (VLO) attenuated chronic morphine-induced behavioral sensitization. The microinjection of TSA blocked the chronic morphine-induced decrease of NF-L. However, our chromatin immunoprecipitation (ChIP)-qPCR results indicated that this effect was not due to the acetylation of histone H3-Lysine 9 and 14 binding to the *NF-L* promotor. In line with the behavioral phenotype, the microinjection of TSA also blocked the chronic morphine-induced increase of p-ERK/p-CREB/p-NF-L. Finally, we compared chronic and acute morphine-induced behavioral sensitization. We found that although both chronic and acute morphine-induced behavioral sensitization were accompanied by an increase of p-CREB/p-NF-L, TSA exhibited opposing effects on behavioral phenotype and molecular changes at different addiction contexts. Thus, our findings revealed a novel role of NF-L in morphine-induced behavioral sensitization, and therefore provided some correlational evidence of the involvement of NF-L in opiate addiction.

## 1. Introduction

Chronic exposure to addictive drugs causes long-lasting changes in the reward-related brain regions. Repeated exposure to drugs of abuse enhances the motor-stimulant response to these drugs, a phenomenon termed behavioral sensitization. In rodents, behavioral sensitization is characterized by more robust psychomotor development following the same or smaller dose of drug challenge after repeated exposure to drugs. Behavioral sensitization involves functionally distinct phases, such as the development phase, transfer phase and expression phase, and specific brain regions may drive these different phases [1,2]. The ventrolateral orbital cortex (VLO), a major area within the orbitofrontal cortex (OFC), has been implicated in morphine-related addictive phenotypes [3]. Bilateral VLO lesions suppress the expression phase of morphine-induced behavioral sensitization [4], and pharmacological manipulation of histone acetylation in the VLO results in dramatic molecular and locomotive alteration [5]. Therefore, epigenetic modulation in the VLO plays a critical role in morphine-induced behavioral sensitization. The extracellular signal-regulated kinases (ERKs) consist of ERK1 and ERK2, and belong to the mitogen-activated protein kinase (MAPK) family. These kinases function throughout the mesolimbic system and are closely related with drug-induced synaptic plasticity. The activity of the transcription factor, cAMP response element binding protein (CREB), is regulated by phosphorylation, and thus could be activated to modulate gene expression through p-ERK. Previous studies proposed that the ERK/CREB signaling pathway may contribute to drug addiction [6]. Pioneering work in our lab has demonstrated that the microinjection of TSA into the VLO promoted acute morphine-induced behavioral sensitization and an acute morphine-induced increase of p-ERK [4]. Chronic morphine abuse is more prevalent, yet the role of VLO on chronic morphine-induced behavioral sensitization remains largely unclear.

Neurofilament (NF) proteins are major components of the neuronal cytoskeleton, and are composed of light subunit (NF-L), medium subunit (NF-M) and heavy subunit (NF-H), according to their molecular weight. The phosphorylation of NF-L proteins is relevant to NF polymerization, dynamic cytoskeletal structure construction and synaptic activity [7]. Due to such essential functions, most recent studies have highlighted that increased plasma and cerebrospinal fluid NF-L is a biomarker for neurodegenerative disorders [8,9]. Moreover, a previous study reported a marked decrease of NF-L proteins in the frontal cortex of chronic opiate abusers [10]. However, the biological function of NF-L in drug addiction remains elusive.

Epigenetic regulation is a key factor in drug-induced adaptations, and the posttranslational modification of histones results in the alteration of gene expression in the brain’s reward circuitry [11]. The modification processes of N-tails of histones lysine (K) residues are dynamically and reversibly catalyzed by histone acetyltransferases (HATs) and histone deacetylases (HDACs), which were involved in mediating neuroadaptations to drug exposure [12]. It has been reported that histone acetylation specific in the vicinity of the NF-L promotor remarkably correlated with NF-L expression [13], and thus endows NF-L with vulnerability to HDAC. Trichostatin A (TSA), a pan HDAC inhibitor, could upregulate acetylated histone H3-Lysine 9 and 14 (H3K9/14ac) in the promoter region of target genes to promote transcriptional activation [14,15].

Here, we used a chronic morphine-induced behavioral sensitization paradigm to investigate the potential role of NF-L in morphine-induced behavioral sensitization. It is noteworthy that the behavioral changes are associated with p-ERK/p-CREB/p-NF-L, but not with direct acetylation at the relevant NF-L loci. These observations demonstrated a correlation of p-NF-L with morphine-induced behavioral sensitization. Further, we provided evidence for the opposing effects of TSA in acute and chronic morphine-induced behavioral sensitization in mice. Such epigenetic regulatory mechanisms might be harnessed for the diagnosis and treatment of opiate addiction in the clinic.

## 2. Results

### 2.1. Microinjection of TSA into the VLO Suppresses the Locomotor Activity during Expression Phase, but Does Not Affect the Locomotor Activity during Development Phase

**Figure 1:**
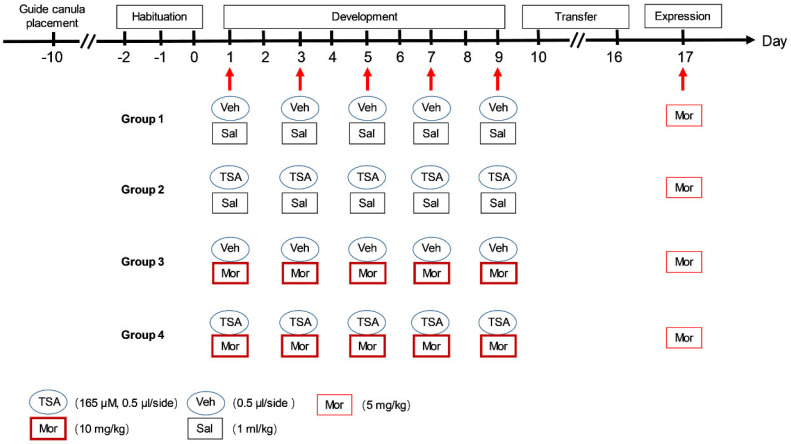
The experimental schedule of chronic morphine-induced behavioral sensitization. Each rat was habituated to the testing chambers without injection from Day 2 to Day 0. During development, they received morphine (10 mg/kg, i.p.) or saline every other day, and their locomotor activity was monitored for 240 min. After 7 days (transfer phase) drug free, all rats received morphine (5 mg/kg, i.p.) and their locomotor activity was measured for 240 min in the expression phase on Day 17.

**Figure 2:**
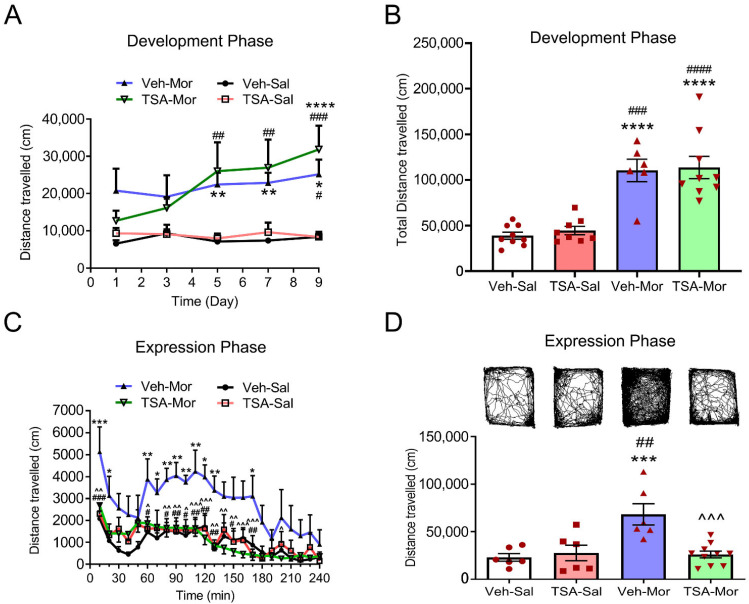
The effects of microinjection of TSA into the VLO on development and expression phases of chronic morphine-induced behavioral sensitization. (**A**) The TSA has no effect on the total travelled distance recorded on Day 1, Day 3, Day 5, Day 7 and Day 9 during development phase. (**B**) The sum of total travelled distance recorded during development phase. (**C**) The TSA reversed morphine-induced hyperlocomotion during expression phase. The travelled distance after morphine (5 mg/kg) challenge was recorded every 10 min within 240 min. (**D**) The sum of total travelled distance of rats for 240 min on Day 17. * *p* < 0.05, ** *p* < 0.01, *** *p* < 0.001, **** *p* < 0.0001, compared with Veh-Sal group; ^#^ *p* < 0.05, ^##^ *p* < 0.01, ^###^ *p* < 0.001, ^####^ *p* < 0.0001, compared with TSA-Sal group; ^ *p* < 0.05, ^^ *p* < 0.01, ^^^ *p* < 0.001, compared with Veh-Mor group. n = 6–10. Abbreviations: Mor, morphine; Sal, saline.

The procedures of chronic morphine-induced behavioral sensitization were show in Figure 1. As shown in Figure 2A, in the development phase of chronic morphine-induced behavioral sensitization, two-way repeated measures ANOVA showed the significant effect of treatment (F_(3, 140)_ = 22.26, *p* < 0.0001), but non-significant effects of interaction (F_(12, 140)_ = 1.120, *p* = 0.3485) and time (F_(4, 140)_ = 1.574, *p* = 0.1846). On Day 5 and Day 7, morphine treatment (10 mg/kg) significantly increased the total distance of rats (Veh-Mor vs. Veh-Sal, *p* < 0.01; TSA-Mor vs. TSA-Sal, *p* < 0.01). On Day 9, the total distance travelled was dramatically different between morphine and saline groups (Veh-Mor vs. Veh-Sal, *p* < 0.05; Veh-Mor vs. TSA-Sal, *p* < 0.05; TSA-Mor vs. TSA-Sal, *p* < 0.001; TSA-Mor vs. Veh-Sal, *p* < 0.0001). However, there is no significant difference in the TSA-Mor group. This trending increase in the TSA-Mor group may reflect some incubation of behavioral sensitization. As shown in Figure 2B, there was no significant difference between the TSA-Morphine group and the Vehicle-Morphine group in the total distance for the whole development phase. In conclusion, the TSA microinjection (165 μM, 0.5 μL/side) has no significant effect during the development phase of morphine-induced behavioral sensitization.

In the expression phase of behavioral sensitization induced by chronic morphine, the total distance of different time points within 240 min were compared and analyzed. As shown in Figure 2C, two-way repeated measures ANOVA revealed the significant effect of time (F_(23, 552)_ = 7.380, *p* < 0.0001) and treatment (F_(3, 552)_ = 73.91, *p* < 0.0001), but not interaction (F_(69, 552)_ = 0.6787, *p* = 0.9768). Further, there was a significant difference in the total distance travelled in 240 min among Veh-Sal, TSA-Sal, Veh-Mor and TSA-Mor groups (F_(3, 24)_ = 9.384, *p* = 0.0003). Morphine significantly increased the total distance of rats (Veh-Mor vs. Veh-Sal, *p* < 0.001; Veh-Mor vs. TSA-Sal, *p* < 0.01). The total distance in the TSA-Morphine group was lower than that of the Vehicle-Morphine group (*p* < 0.001, Figure 2D). These results suggested that the bilateral microinjection of TSA (165 μM, 0.5 μL/side) into the VLO significantly suppressed morphine-induced behavioral sensitization.

### 2.2. Chronic Morphine-Induced Behavioral Sensitization Inhibits the Expression of Total NF-L in the VLO, While TSA Microinjection Reverses This Inhibitory Effect

**Figure 3:**
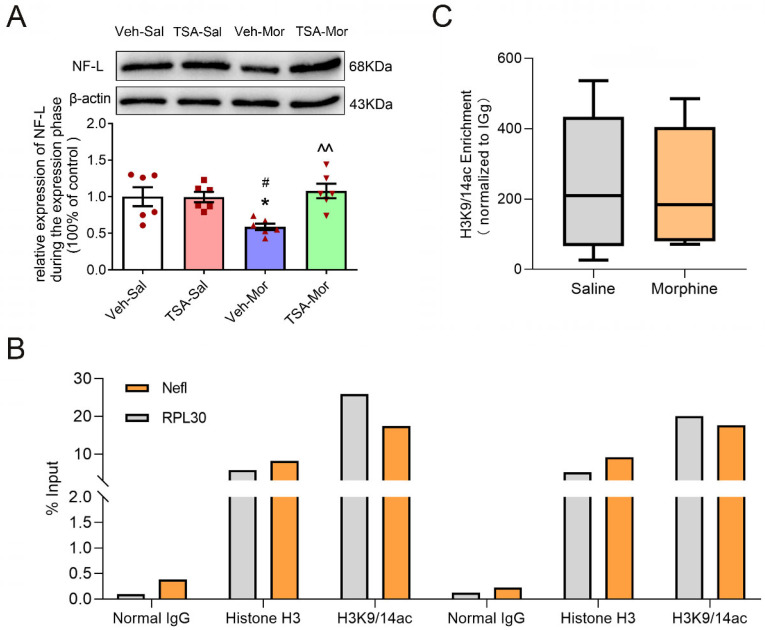
Microinjection of TSA into the VLO blocked chronic morphine-induced downregulation of NF-L, and chronic morphine-induced alteration of NF-L was not due to H3K9/14ac binding to *NF-L* promotor. (**A**) Representative Western blots (top) and summary scatterplots with histograms (bottom) demonstrated the protein levels of NF-L in the VLO. * *p* < 0.05, compared with Veh-Sal group; ^#^ *p* < 0.05, compared with TSA-Sal group; ^^ *p* < 0.01, compared with Veh-Mor group. n = 6. (**B**) The Ct data from different IPs was calculated corresponding 2% input sample. Fold enrichment levels were normalized with their respective control IgG values. (**C**) No significant difference of enrichment of H3K9/14ac at the *NF-L* promoter region between morphine and saline groups. n = 6.

The protein level of NF-L significantly altered during the expression phase of chronic morphine-induced behavioral sensitization. As shown in Figure 3, the expression level of NF-L was significantly different among Veh-Sal, TSA- Sal, Veh-Mor and TSA-Mor groups (F_(3, 20)_ = 5.877, *p* = 0.0048). The expression level of NF-L was dramatically decreased in the Veh-Mor group compared with the Veh-Sal group (*p* < 0.05). There was no significant difference in the expression level of NF-L between the TSA-Mor group and TSA-Sal group, while the expression level of NF-L in the TSA-Mor group was significantly higher than that of the Veh-Mor group (*p* < 0.01). These results suggested that the bilateral microinjection of TSA into the VLO could reverse the down-regulation of NF-L expression after chronic morphine-induced behavioral sensitization.

### 2.3. The Down-Regulation of NF-L Expression after Chronic Morphine-Induced Behavioral Sensitization Is Not Due to the Direct Acetylation of H3K9/14 at NF-L Gene

Previous studies have demonstrated that TSA, a pan HDAC inhibitor, functions by acetylating H3K9/14ac at the promoter region of target genes [14,15]. To determine the mechanisms of NF-L on morphine-induced behavioral sensitization, we used ChIP to measure the enrichment H3K9/14ac at *NF-L* gene. As shown in Figure 3B, the % Input of RPL30 (positive control) was greater than 1%, while the % input of IgG-negative control was less than 0.1%. The relative enrichment abundance of the H3K9/14ac at the promoter region of rat NF-L was more than 10 times higher than that of IgG control. These results verified our experimental quality control. As shown in Figure 3C, there was no significant difference of H3K9/14ac enrichment at *NF-L* promoter region between morphine and saline groups (t_10_ = 0.1239, *p* = 0.903), suggesting that the morphine-induced alteration of NF-L was not due to the direct effect of H3K9/14ac.

### 2.4. Chronic Morphine-Induced Behavioral Sensitization Promotes the Phosphorylation of ERK, CREB and NF-L in the VLO, While TSA Microinjection Reverses These Effects

**Figure 4:**
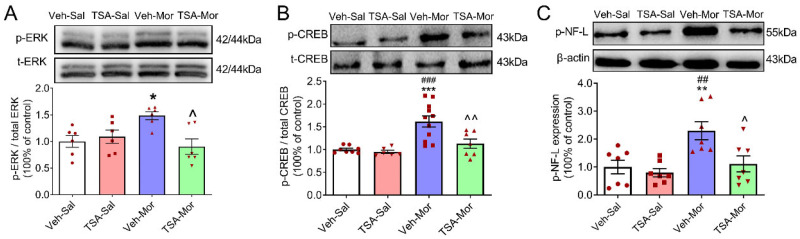
Microinjection of TSA into the VLO blocked chronic morphine-induced upregulation of p-ERK, p-CREB and p-NF-L. (**A**) Representative Western blots (top) and summary scatterplots with histograms (bottom) demonstrate the protein levels of p-ERK/t-ERK in the VLO. (**B**) Representative Western blots (top) and summary scatterplots with histograms (bottom) demonstrate the protein levels of p-CREB/t-CREB in the VLO. (**C**) Representative Western blots (top) and summary scatterplots with histograms (bottom) demonstrate the protein levels of p-NF-L in the VLO. * *p* < 0.05, ** *p* < 0.01, *** *p* < 0.001, compared with Veh-Sal group; ^##^ *p* < 0.01, ^###^ *p* < 0.001, compared with TSA-Sal group; ^ *p* < 0.05, ^^ *p* < 0.01, compared with Veh-Mor group. n = 6–11.

Mounting evidence has demonstrated that ERK and CREB are important for opiate addiction [16,17]. There were significant differences in the protein level of *p*-ERK among Veh-Sal, TSA-Sal, Veh-Mor and TSA-Mor groups (F_(3, 20)_ = 4.824, *p* = 0.01) after the challenge dose of morphine exposure on Day 17. The p-ERK expression significantly increased in the Veh-Mor group, compared with the Veh-Sal group, while the microinjection of TSA into the VLO significantly decreased the protein levels of p-ERK (Veh-Sal vs. TSA-Sal, *p* > 0.05; Veh-Sal vs. Veh-Mor, *p* < 0.05; Veh-Sal vs. TSA-Mor, *p* > 0.05; TSA-Sal vs. Veh-Mor, *p* > 0.05; TSA-Sal vs. TSA-Mor, *p* > 0.05; Veh-Mor vs. TSA-Mor, *p* < 0.05). There was no significant difference of total ERK among these groups (Figure 4A).

There were significant differences in the protein level of p-CREB among Veh-Sal, TSA-Sal, Veh-Mor and TSA-Mor groups (F_(3, 28)_ = 11.25, *p* < 0.0001) after the challenge dose of morphine exposure on Day 17. The protein levels of p-CREB significantly increased in the expression phase of morphine-induced behavioral sensitization, while the microinjection of TSA into the VLO significantly decreased the protein levels of p-CREB (Veh-Sal vs. TSA-Sal, *p* > 0.05; Veh-Sal vs. Veh-Mor, *p* < 0.001; Veh-Sal vs. TSA-Mor, *p* > 0.05; TSA-Sal vs. Veh-Mor, *p* < 0.001; TSA-Sal vs. TSA-Mor, *p* > 0.05; Veh-Mor vs. TSA-Mor, *p* < 0.01). There was no significant difference of total CREB among these groups (Figure 4B).

There were significant differences in the protein level of p-NF-L among Veh-Sal, TSA- Sal, Veh-Mor and TSA-Mor groups (F_(3, 24)_ = 6.919, *p* = 0.0016) after the challenge dose of morphine exposure on Day 17. The protein levels of p-NF-L significantly increased in the expression phase of morphine-induced behavioral sensitization, while the microinjection of TSA into the VLO significantly decreased the protein levels of p-NF-L (Veh-Sal vs. TSA-Sal, *p* > 0.05; Veh -Sal vs. Veh-Mor, *p* < 0.01; Veh-Sal vs. TSA-Mor, *p* > 0.05; TSA-Sal vs. Veh-Mor, *p* < 0.01; TSA-Sal vs. TSA-Mor, *p* > 0.05; Veh-Mor vs. TSA-Mor, *p* < 0.05, Figure 4C).

The results of the p-ERK protein and p-CREB protein may suggest that the activation of ERK and CREB is correlated with chronic morphine-induced behavioral sensitization.

### 2.5. Acute Morphine-Induced Behavioral Sensitization Promotes the Phosphorylation of CREB and NF-L in the VLO, While TSA Microinjection Reinforces These Effects

**Figure 5:**
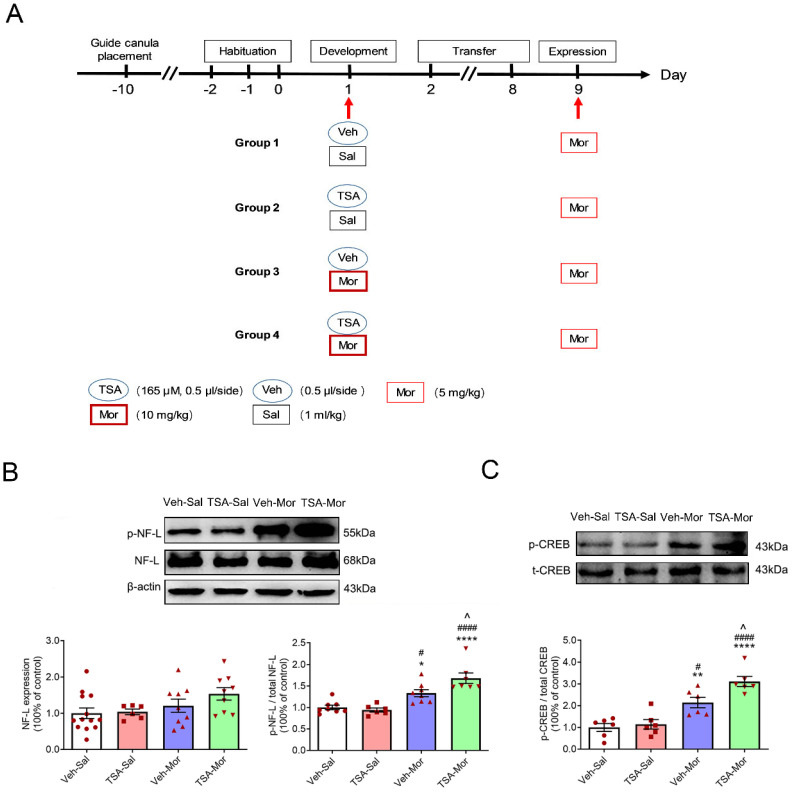
Microinjection of TSA into the VLO reinforced acute morphine-induced upregulation of p-NF-L and p-CREB. (**A**) The experimental schedule of acute morphine-induced behavioral sensitization. During development, they received single dose of morphine (10 mg/kg, i.p.). After 7 days (transfer phase) drug free, all rats received morphine (5 mg/kg, i.p.) and their locomotor activity was measured for 240 min in the expression phase on Day 9. (**B**) Representative Western blots (top) and summary scatterplots with histograms (bottom) demonstrate the protein levels of p-NF-L in the VLO. Representative Western blots and summary scatterplots with histograms demonstrate the protein levels of total NF-L in the VLO. (**C**) Representative Western blots (top) and summary scatterplots with histograms (bottom) demonstrate the protein levels of p-CREB in the VLO. * *p* < 0.05, ** *p* < 0.01, **** *p* < 0.0001, compared with Veh-Sal group; ^#^ *p* < 0.05, ^####^ *p* < 0.0001, compared with TSA-Sal group; ^ *p* < 0.05, compared with Veh-Mor group. n = 6–13.

The experimental schedule of acute morphine-induced behavioral sensitization was shown in Figure 5 as previously reported [4]. During the development phase, rats received a single dose of morphine (10 mg/kg). After 7 days (transfer phase) drug free, all rats received morphine (5 mg/kg) and their locomotor activity was measured for 240 min in the expression phase on Day 9. Our previous study has demonstrated that the microinjection of TSA into the VLO potentiated acute morphine-induced behavioral sensitization and the acute morphine-induced increase of p-ERK [4]. There was no significant difference in the protein level of NF-L among Veh-Sal, TSA- Sal, Veh-Mor and TSA-Mor groups (F_(3,33)_ = 2.286, *p* = 0.0969) after the challenge dose of morphine exposure on Day 9 (Figure 5A).

As shown in Figure 5A, the expression level of phosphorylated NF-L protein (p-NF-L) has a significant difference among Veh-Sal, TSA- Sal, Veh-Mor and TSA-Mor groups (F_(3, 25)_ = 16.40, *p* < 0.0001). The expression level of p-NF-L was significantly increased after morphine exposure (Veh-Mor vs. Veh-Sal, *p* < 0.05; Veh-Mor vs. TSA-Sal, *p* < 0.05; TSA-Mor vs. TSA-Sal, *p* < 0.0001; TSA-Mor vs. Veh-Sal, *p* < 0.0001). The bilateral microinjection of TSA into the VLO further upregulated the protein level of p-NF-L (TSA-Mor vs. Veh-Mor, *p* < 0.05).

**Figure 6:**
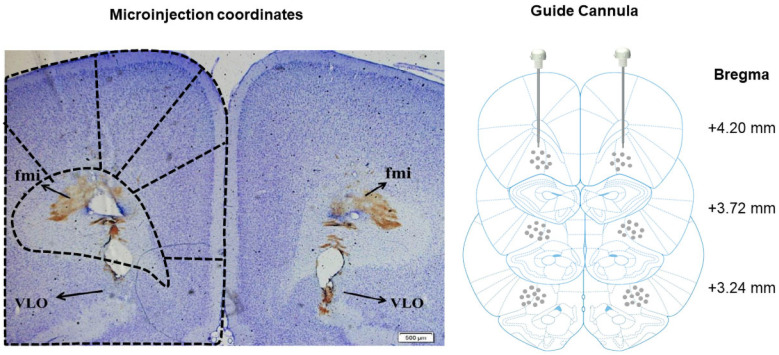
A representative image of a coronal brain section showing the localization of VLO guide cannulas insertion and microinjection. The reference coordinates: anterior-posterior (AP) + 3.2; medial-lateral (ML) ± 2.0; dorsal-ventral (DV) 2.6 mm.

As shown in Figure 5B, there was a significant difference in p-CREB protein levels (F_(3, 20)_ = 20.18, *p* < 0.0001). The expression level of p-CREB was significantly increased after morphine exposure, and TAS treatment enhanced this effect (Veh-Mor vs. Veh-Sal, *p* < 0.01; Veh-Mor vs. TSA- Sal, *p* < 0.05; TSA-Mor vs. TSA-Sal, *p* < 0.0001; TSA-Mor vs. Veh-Sal, *p* < 0.0001; TSA-Mor vs. Veh-Mor, *p* < 0.05). There was no significant difference of total CREB among these groups (*p* > 0.05). The injection sites were historically identified and an example was shown in Figure 6.

These results suggested that previous exposure to acute morphine resulted in an increase of p-CREB/p-NF-L in the VLO, and the single microinjection of TSA further enhanced this effect.

## 3. Discussion

Gender differences are apparent throughout different stages of drug abuse [18]. Therefore, only male rats were used in this study to exclude the complex reciprocal interactions of estrous phases with the brain in female animals.

### 3.1. Microinjection of TSA into the VLO Significantly Decreased Rats’ Locomotion in the Expression Phase of Morphine-Induced Chronic Behavioral Sensitization

Rats that were exposed to morphine showed an initial inhibition of locomotion activity for approximately 60 min and then a robust increase of locomotion activity later [19]. Consistent with previous studies [20,21,22], the present study indicated that repeated and intermittent morphine treatment (10 mg/kg), followed by a challenge dose of 5 mg/kg, was able to induce the expression of behavioral sensitization in rats. While HDAC inhibitor TSA into the VLO had no effect during the development phase, it depressed the expression phase of morphine-induced behavioral sensitization (Figure 2).

In the context of aversive instrumental learning, previous research has shown that the inactivation of the VLO via the GABA agonists baclofen and muscimol did not impair initial learning or acquisition, but increased the expression once stimulus-outcome associations had been well established [23]. Partly consistent with these findings, our previous data demonstrated that the bilateral electrical lesion of the VLO has no effect on the development phase or initial learning, but significantly suppressed the expression of morphine-induced behavioral sensitization [4]. Collectively, the above evidence supported that compared with the initial learning process, the VLO is more important during the behavioral expression phase. Specific brain regions may modulate different phases of behavioral sensitization, which could at least partly illustrate the different effects of TSA during different phases. Moreover, TSA pre-treatment blocked the increase of p-ERK/ERK, p-CREB/CREB ratios and p-NF-L, and these molecular changes might contribute to the blocking effect of TSA during the expression phase. Additionally, different effects may be observed depending on the stimulus valance, i.e., aversive versus rewarding. The rewarding stimulus might be preferentially endorsed by the VLO, while the inhibition of VLO promoted aversive responses.

### 3.2. The Inhibitory Effect of TSA on Chronic Morphine-Induced Behavioral Sensitization Might Be Correlated with p-ERK/p-CREB/p-NF-L Signaling Pathway, Rather Than Having a Direct Influence on NF-L Expression

Mounting evidence suggested that histone marks, such as H3K14ac, could promote chromatin relaxation and gene expression, which are essential for learning and memory [24]. Alcohol exposure in mice resulted in the significant genome-wide enrichment of H3K9ac peaks at key neuronal genes [25]. Subbanna et al. also reported that the alteration of H3K14ac in the activity-regulated cytoskeletal (Arc) gene promoter in the hippocampus is responsible for alcohol spectrum disorders [26]. At the same time, ethanol exposure enhanced the repressive marker, such as H3K9me2, due to the enhanced recruitment of methyltransferase G9a, accompanied by defects in hippocampal synaptic plasticity and the disruption of spatial memory, while the G9a inhibitor dramatically reversed these negative effects. Collectively, these findings validated that histone modifications could drive long-lasting adaptations to cellular function via cytoskeletal proteins, which promotes the behavioral and psychological abnormalities underlying addiction.

A previous study reported that the TSA, which is a pan HDAC inhibitor, enhanced histone H3-Lysine 9 and 14 acetylation (H3K9/14ac) at promoter regions of target genes [14,15]. As histone deacetylases are involved in chromatin compaction and mostly histone acetylation is correlated with transcriptional permission, TSA could promote transcription. The NF-L is a well-known marker for axonal–neuronal degeneration. Most studies reported a marked increase of NF-L in neurodegenerative diseases, but we detected a significant downregulation of total NF-L after chronic morphine exposure and TSA reversed this effect. Similar to the study reported by García-Sevilla [27], chronic morphine treatment decreased the immune-density of NF-L in the cerebral cortex. These abnormalities in NF proteins turned out to be related to opioid receptors, for the chronic morphine treatment did not decrease NF-L in cortices of µ-, δ-, and κ-knockout mice, suggesting the involvement of the three types of opioid receptors in this effect of morphine. We further examined the recruitment of H3K9/14ac at *NF-L* promoters via the ChIP approach, but did not observe any difference, excluding a direct causal link between histone acetylation and *NF-L*. Future studies using ChIP, in combination with high-throughput PCR sequencing to identify the direct downstream target genes, is warranted.

The ERK plays an Important role in long-term synaptic plasticity [28], and the increase of ERK phosphorylation in the prefrontal cortex, nucleus accumbens, hippocampus and amygdala is involved in morphine addiction in mice [29,30]. The p-EKR and p-CREB proteins in the VLO were upregulated after morphine exposure, which derives from an epigenetic regulation, as this effect could be blocked by repeated TSA injection. Similarly, as a transcription factor, CREB is particularly implicated in the plasticity associated with drug addiction. Previous study showed that ERK could conduct the transcriptional regulation by controlling the phosphorylation of CREB via mitogen- and stress-activated protein kinase 1 (MSK1) [31,32]. Another study also demonstrated that conditioned place aversion (CPA) extinction training-induced chromatin modification of BDNF in the PFC is trigged by the activation of ERK-CREB signaling pathways [13]. Therefore, ERK-CREB signaling pathways could be epigenetically regulated.

The activation of ERK is vital for morphine-induced behavioral sensitization. In the expression phase of morphine-induced behavioral sensitization, CREB can be phosphorylated by ERK, via an opioid receptor, to influence the transcription and expression of the NF-L gene (regulated by p-CREB) [27]. Consistent with our speculation that NF-L proteins could be phosphorylated by ERK, TSA blocked the morphine-induced increase of p-NF-L and p-ERK.

### 3.3. The TSA Has Opposing Effects on Acute versus Chronic Morphine-Induced Behavioral Sensitization

**Figure 7:**
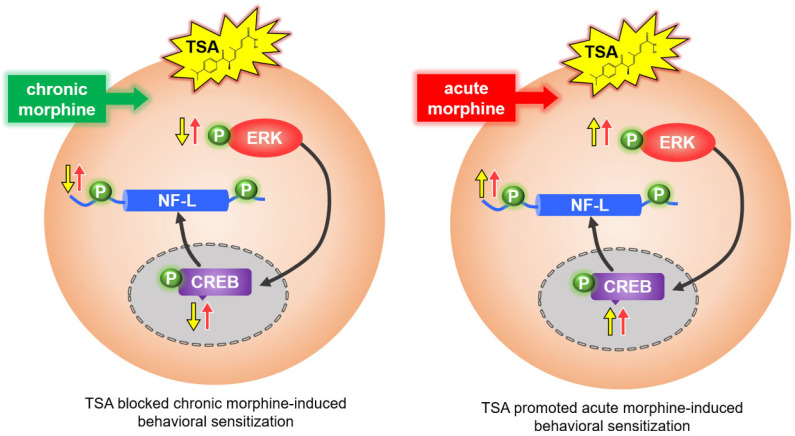
A schematic graph showing that TSA displayed opposing effects on chronic and acute morphine-induced behavioral sensitization. Microinjection of TSA into the VLO blocked chronic morphine-induced behavioral sensitization, which is correlated with p-ERK/p-CREB/p-NF-L pathway. However, microinjection of TSA into the VLO promoted acute morphine-induced behavioral sensitization.

Our previous study demonstrated that the microinjection of TSA into the VLO potentiated acute morphine-induced behavioral sensitization and the increase of p-ERK [4], and our current study further validated that TSA also elevated the acute morphine-induced increase of p-CREB and p-NF-L (Figure 5). These findings are very interesting, as TSA displayed opposing effects on chronic and acute morphine-induced behavioral sensitization (Figure 7). The changes observed in the chronic morphine groups need time to accumulate, as a single injection of morphine is not enough to induce the dramatic alteration of NF-L (Figure 5A). The injection times, i.e., single injection of TSA versus repeated TSA injection throughout the development phase, might partially explain the discrepancy. Moreover, this result is reminiscent of other VLO studies, in which the microinjection of the 5-HT6 receptor agonist into the VLO aggregated acute inflammatory pain and attenuated chronic neuropathic pain [33,34]. Different cell types, such as glutamatergic versus GABAergic neurons, and dopamine D1 receptor–expressing (D1R) neurons versus D2R neurons, exhibited different, and even opposing effects on drug-related behaviors. We speculated that cell type–specific mechanisms may drive the different effects of TSA on chronic versus acute morphine-induced behavioral sensitization. Furthermore, different contexts, such as acute versus chronic morphine exposure, induced dramatically different neuroplasticity changes. This is also the case with distinct phases of behavioral sensitization. For example, the development phase implicates transient neuronal maladaptive alterations, while the expression phase indicates time-dependent neuronal adaptive changes evoked by drug re-exposure. Using optogenetic or chemogenetic strategies to manipulate specific types of neurons, ongoing research efforts will focus on comparing and dissecting the molecular underpinnings of acute and chronic morphine-related behaviors.

One limitation of the current study is that most data in this study are correlational, which limits our ability to explain the causal relationship. We cannot rule out other factors such as receptor phosphorylation, receptor expression and other synaptic/circuit mechanisms by which behavioral sensitization may be influenced. Importantly, we did not investigate the direct target genes on which TSA exerted its activity. Although mounting evidence has established that TSA could affect phosphorylation of ERK [35,36,37,38,39] and CREB [15,40], the underlying mechanisms remain unknown. In order to further advance our understanding, it is warranted to utilize other assays, such as ChIP-sequencing, to identify direct target genes. Additionally, we should incorporate target gene manipulation strategies to identify their functions.

In conclusion, the present study suggests that NF-L in the VLO is correlated with morphine-induced behavioral sensitization. TSA regulates morphine-induced behavioral sensitization, which is correlated with p-ERK/p-CREB/p-NF-L pathway in the VLO, rather than by previously reported H3K9/14 binding to the NF-L promotor. In addition, acute and chronic morphine exposure produced similar adaptations in the VLO, while TSA produced very different adaptations. Thus, our data provided some insights for the diagnosis and treatment of opiate addiction in the clinic.

## 4. Materials and Methods

### 4.1. Animals and Drugs

Male adult Sprague-Dawley rats (weighing 250–300 g) were purchased from the Medical Experimental Animal Center of Xi’an Jiaotong University, Shaanxi Province, China. Five animals were housed in each cage under a 12/12 h light-dark cycle (lights on at 07:00) with ad libitum to food and water. The experimental protocols were approved by the Institutional Animal Care Committee of Xi’an Jiaotong University (No. 2015491). All efforts were made to minimize the number of rats used and their suffering in the experiments.

Morphine hydrochloride was purchased from the National Institutes for Food and Drug Control (Beijing, China), which was dissolved in 0.9% saline and administered intraperitoneally (i.p.) [19]. The Trichostatin A (TSA) was purchased from Sigma-Aldrich (Sigma Chemical, St. Louis, MO, USA) and dissolved in 10% dimethylsulfoxide (DMSO, diluted with saline) to obtain a solution of 165 μM [13,15,41]. All drugs were freshly prepared before the experiments.

### 4.2. Surgery for Bilateral Intracerebral Guide Cannula Placement

Rats were anesthetized with sodium pentobarbital (50 mg/kg, i.p.), and the head was immobilized in a stereotaxic frame. A small craniotomy was performed just above the VLO. Stainless steel guide cannulas were stereotaxically inserted, with the tip 2.0 mm dorsal to the bilateral VLO using the following coordinates: anterior-posterior (AP) + 3.2; medial-lateral (ML) ± 2.0; dorsal-ventral (DV) 2.6 mm [42,43], followed by attachment to the skull with two microscrews and dental cement. Once the rats recovered from anesthesia, they were administered with sodium penicillin (0.2 million units/day for 3 days, i.p.) to prevent infection. The rats were carefully nursed and fed in clean cages. After surgery, animals were housed separately in cages and allowed 7 days to recover before behavioral procedures.

### 4.3. Intracerebral Microinjection

A microsyringe was inserted into the VLO, with the tip extending 2.0 mm beyond the end of the guide cannula from the surface of brain. TSA (0.5 μL/side) was slowly infused into the VLO bilaterally through a 1.0-μL Hamilton syringe at a constant speed over 1 min to make sure the drugs completely diffused from the tips, and then the obturators were reinserted into the guide cannula [4]. Equal volumes of 10% DMSO were served as the vehicle controls.

### 4.4. Behavioral Sensitization

For behavioral experiments, rats were randomly assigned to each of four groups, i.e., Vehicle-Saline (Veh-Sal), TSA-Saline (TSA-Sal), Vehicle-Morphine (Veh-Mor) and TSA-Morphine (TSA-Mor). The locomotor activity test system consists of four testing chambers (60 × 60 × 40 cm^3^, length × width × height) with a video camera above to track each animal’s locomotion. The room was dimly lit and the whole experiments were performed between 8:00 am and 12:00 noon. The procedures were performed according to a previous report with some modifications [4]. Before the experiment, each rat was habituated to the testing chambers for 3 days from Day 2 to Day 0. From Day 1 to Day 9 (development phase), morphine (10 mg/kg, i.p.) or saline (1 mL/kg, i.p.) was injected every other day, 5 times in total. From Day 10 to Day 16 (transfer phase), all groups were kept in their home cages drug free for 7 days. All rats were challenged with a low dose of morphine (5 mg/kg) on Day 17 (expression phase). Following the morphine or saline injections, horizontal locomotor activity was monitored for 240 min, which was recorded at 10 min intervals (Figure 1). For acute or a single dose of morphine-induced sensitization, 10 mg/kg of morphine was injected only on Day 1, followed by 7 days of transfer, and challenged on Day 9 (Figure 5A) [4]. The microinjection of TSA (165 μM, 0.5 μL/side) or vehicle was slowly infused into the VLO bilaterally 30 min prior to morphine or saline. Horizontal trajectories (reflecting locomotor activity) of the rats were recorded and analyzed off-line by a video-tracking software (SMART, Panlab SL, Barcelona, Spain) to determine their total travelled distance. Animals were sacrificed immediately after the 240 min locomotor activity test for molecular experiments.

### 4.5. Western Blotting

After behavioral experiments, 13 rats in each group were sacrificed directly and the whole brain was removed. Then the brain was cut into coronal sections with 1 mm thickness and the location of microinjection was checked. The VLO tissue will be extracted for Western blot analysis only from rats with accurate microinjection site. Protein extraction was performed as previously described [19]. Briefly, proteins were separated on SDS-PAGE gels and then transferred to PVDF membranes (Millipore Corporation, Bedford, MA, USA). The membranes were blocked in 5% (*w*/*v*) skimmed milk in Tris-buffered saline (500 mM NaCl, 20 mM Tris–HCl, pH 7.5) containing 0.05% Tween-20 for 1 h at room temperature and then incubated with the following primary antibodies overnight at 4 °C: phosphorylation (p)-NF-L at Ser^55^ (sc-24504, Santa Cruz Biotechnology, Santa Cruz, CA, USA), NF-L (#2837, Cell Signaling Technology, Danvers, MA, USA) at 1:1000, p-ERK1/2 (ab201015, Abcam, Cambridge, UK), total ERK1/2 (#9102, Cell Signaling Technology, Danvers, MA, USA) at 1:4000, p-CREB at Ser^133^ (#9198, Cell Signaling Technology), total CREB (#9197, Cell Signaling Technology, Danvers, MA, USA) at 1:1000, and β-actin (sc-47778, Santa Cruz) at 1:1000. The membranes were rinsed 3 times and then incubated with secondary antibodies: goat anti-rabbit or anti-mouse IgG horse-radish peroxidase (HRP)-conjugated secondary antibody (BS13278 & BS12478, Bioworld, Dublin, OH, USA) at 1: 10,000. Development was performed with an enhanced chemiluminescence (ECL) plus a detection kit (Millipore Corporation, Bedford, MA, USA). The band intensities were analyzed using the Quantity One software (Bio-Rad, Hercules, CA, USA) to calculate the protein versus the loading control (β-actin) for each protein.

### 4.6. Chromatin Immunoprecipitation (ChIP)-qPCR Assay

ChIP assays were performed using the Simple ChIP Plus Enzymatic Chromatin IP Kit (#9005, Cell Signaling Technology) according to the manufacturer’s protocol with minor modifications. Brain tissues containing VLO were collected from animals on Day 17 immediately after the behavioral test. Four samples were pooled for cross-linking.

Weigh the fresh tissue samples and place samples in a 60 mm dish and finely mince using a clean razor blade on ice. To crosslink proteins to DNA, add 45 μL of 37% formaldehyde and rock for 20 min at room temperature. Stop cross-linking by adding 100 μL of 10× *g* and mix for 5 min at room temperature. After centrifugation, transfer tissue and re-suspend in PBS to a Dounce homogenizer, and then disaggregate tissue pieces with 20–25 strokes to make single-cell suspension. Remove supernatants and continue with chromatin digestion. Chromatin was digested with micrococcal nuclease (#10011, Cell Signaling Technology) for 20 min at 37 °C with frequent mixing to digest DNA into a length of approximately 150–900 bp. The clarified nuclear extracts were incubated at 4 °C with anti-acetyl H3K9/14 (#9677, Cell Signaling Technology), Histone H3 (#4620, Cell Signaling Technology) and Normal Rabbit IgG (#2729, Cell Signaling Technology), with the latter two as the positive and negative control, respectively. Following an overnight incubation with antibodies, 30 μL of Protein G Magnetic Beads was added to each IP reaction and incubated for 2 h at 4 °C with rotation. Bound DNA–protein complexes were eluted and crosslinks were reversed after a series of washes. CHIP-enriched DNA was analyzed by quantitative PCR performed in triplicate using Bio-Rad CFX 96 (Bio-Rad, USA), yielding a final sample size of 6 data points.

PCR reactions were performed in a 10-μL system. The primers were designed to include in the vicinity of the *NF-L* promoter with acetylation of histones and the sequences as follows: *NF-L* forward, 5′-CCCGCCTTTGCTCTTGC; *NF-L* reverse, 5′-GGCTGTCCTGCCACCTCTAT. The qPCR conditions were as follows: 95 °C for 30 s (initial denaturation), 40 cycles for 95 °C for 5 s (denaturation), 62 °C for 30 s (annealing) and 95 °C for 10 s followed by Melt Curve 65.0 to 95.0 °C for 5 s. The signal relative to the input was calculated using a formula from the manufacture’s protocol as follows: Percent input = 2% × 2^(C[T]2%input sample−C[T]IP sample)^, where C[T] = Average threshold cycle of PCR reaction [44]. Rat Ribosomal Protein L30 (RPL30) primer was used for positive control in real-time PCR reaction [45,46,47,48]. Fold enrichment levels of the promotor region for *NF-L* was assessed relative to the 2% input DNA followed by normalization to the respective control IgG values.

### 4.7. Histology Examination

Three rats in each group were deeply anesthetized after the behavioral tests and transcardially perfused with 0.9% saline, followed by 10% formalin. Their brains were then removed and fixed in 10% formalin solution for 3–7 days, then stored in 30% sucrose (pH 7.4) overnight. The brains were then cut into 40-μm thick sections using a freezing microtome, and the slices were stained with Cresyl Violet. For animals whose injection sites were not within the VLO, their behavioral data was excluded from data analysis. A representative verification of VLO microinjection was shown in Figure 6.

### 4.8. Data Analysis

All data were shown as mean ± SEM. The statistical analyzes were performed using Student’s *t*-test, one-way ANOVA and two-way ANOVA. For results with significant interaction effects in two-way ANOVA, the simple effect test was conducted for further analysis. For results without significant interaction effects, Bonferroni’s post-hoc test or Student’s *t*-test was conducted. To determine differences between two groups, a Student’s *t*-test was performed. For Western blotting, the protein levels of the control group were set at 100% and all data were normalized to loading control. The data analyzes were performed using IBM SPSS statistics 24. *p* < 0.05 was considered statistically significant.

## Data Availability

The datasets generated during and/or analyzed during the current study are available from the corresponding author upon reasonable request.

## Abbreviations

CREB, cAMP response element-binding protein; ERK, extracellular signal-regulated kinase; HDAC, histone deacetylases; NF-L, neurofilament light subunit; TSA, Trichostatin A; VLO, ventrolateral orbital cortex.

## References

[1] Baumbach J.L., McCormick C.M. (2021). Nicotine sensitization (part 1): Estradiol or tamoxifen is required during the induction phase and not the expression phase to enable locomotor sensitization to nicotine in female rats. Psychopharmacology.

[2] Kozlova A., Butler R.R., Zhang S., Ujas T., Zhang H., Steidl S., Sanders A.R., Pang Z.P., Vezina P., Duan J. (2021). Sex-specific nicotine sensitization and imprinting of self-administration in rats inform GWAS findings on human addiction phenotypes. Neuropsychopharmacology.

[3] Hardung S., Epple R., Jäckel Z., Eriksson D., Uran C., Senn V., Gibor L., Yizhar O., Diester I. (2017). A Functional Gradient in the Rodent Prefrontal Cortex Supports Behavioral Inhibition. Curr. Biol..

[4] Wei L., Zhu Y.-M., Zhang Y.-X., Liang F., Barry D.M., Gao H.-Y., Li T., Huo F.-Q., Yan C.-X. (2016). Microinjection of histone deacetylase inhibitor into the ventrolateral orbital cortex potentiates morphine induced behavioral sensitization. Brain Res..

[5] Wei L., Liu B., Yao Z., Yuan T., Wang C., Zhang R., Wang Q., Zhao B. (2021). Sirtuin 1 inhibitor EX527 suppresses morphine-induced behavioral sensitization. Neurosci. Lett..

[6] Fan L., Chen H., Liu Y., Hou H., Hu Q. (2023). ERK signaling is required for nicotine-induced conditional place preference by regulating neuroplasticity genes expression in male mice. Pharmacol. Biochem. Behav..

[7] Yuan A., Sershen H., Veeranna, Basavarajappa B.S., Kumar A., Hashim A., Berg M., Lee J.H., Sato Y., Rao M.V. (2015). Neurofilament subunits are integral components of synapses and modulate neurotransmission and behavior in vivo. Mol. Psychiatry.

[8] Ashton N.J., Janelidze S., Al Khleifat A., Leuzy A., van der Ende E.L., Karikari T.K., Benedet A.L., Pascoal T.A., Lleó A., Parnetti L. (2021). A multicentre validation study of the diagnostic value of plasma neurofilament light. Nat. Commun..

[9] Yuan A., Nixon R.A. (2021). Neurofilament Proteins as Biomarkers to Monitor Neurological Diseases and the Efficacy of Therapies. Front. Neurosci..

[10] García-Sevilla J.A., Ventayol P., Busquets X., La Harpe R., Walzer C., Guimón J. (1997). Marked decrease of immunolabelled 68 kDa neurofilament (NF-L) proteins in brains of opiate addicts. Neuroreport.

[11] Zhang Y.X., Akumuo R.C., España R.A., Yan C.X., Gao W.J., Li Y.C. (2018). The histone demethylase KDM6B in the medial prefrontal cortex epigenetically regulates cocaine reward memory. Neuropharmacology.

[12] Cadet J.L., Jayanthi S. (2021). Epigenetics of addiction. Neurochem. Int..

[13] Wang W.-S., Kang S., Liu W.-T., Li M., Liu Y., Yu C., Chen J., Chi Z.-Q., He L., Liu J.-G. (2012). Extinction of aversive memories associated with morphine withdrawal requires ERK-mediated epigenetic regulation of brain-derived neurotrophic factor transcription in the rat ventromedial prefrontal cortex. J. Neurosci..

[14] Sagarkar S., Balasubramanian N., Mishra S., Choudhary A.G., Kokare D.M., Sakharkar A.J. (2019). Repeated mild traumatic brain injury causes persistent changes in histone deacetylase function in hippocampus: Implications in learning and memory deficits in rats. Brain Res..

[15] Wang Y., Lai J., Cui H., Zhu Y., Zhao B., Wang W., Wei S. (2015). Inhibition of histone deacetylase in the basolateral amygdala facilitates morphine context-associated memory formation in rats. J. Mol. Neurosci..

[16] Yin F., Zhang J., Lu Y., Zhang Y., Liu J., Deji C., Qiao X., Gao K., Xu M., Lai J. (2022). Modafinil rescues repeated morphine-induced synaptic and behavioural impairments via activation of D1R-ERK-CREB pathway in medial prefrontal cortex. Addict. Biol..

[17] Zhu H., Zhuang D., Lou Z., Lai M., Fu D., Hong Q., Liu H., Zhou W. (2021). Akt and its phosphorylation in nucleus accumbens mediate heroin-seeking behavior induced by cues in rats. Addict. Biol..

[18] Pogun S., Yararbas G., Nesil T., Kanit L. (2017). Sex differences in nicotine preference. J. Neurosci. Res..

[19] Wei L., Zhu Y.-M., Zhang Y.-X., Liang F., Li T., Gao H.-Y., Huo F.-Q., Yan C.-X. (2016). The α1 adrenoceptors in ventrolateral orbital cortex contribute to the expression of morphine-induced behavioral sensitization in rats. Neurosci. Lett..

[20] Kalivas P.W., Duffy P. (1987). Sensitization to repeated morphine injection in the rat: Possible involvement of A10 dopamine neurons. J. Pharmacol. Exp. Ther..

[21] Kuribara H. (1996). Effects of interdose interval on ambulatory sensitization to methamphetamine, cocaine and morphine in mice. Eur. J. Pharmacol..

[22] Ojanen S., Koistinen M., Bäckström P., Kankaanpää A., Tuomainen P., Hyytiä P., Kiianmaa K. (2003). Differential behavioural sensitization to intermittent morphine treatment in alcohol-preferring AA and alcohol-avoiding ANA rats: Role of mesolimbic dopamine. Eur. J. Neurosci..

[23] Jean-Richard-Dit-Bressel P., McNally G.P. (2016). Lateral, not medial, prefrontal cortex contributes to punishment and aversive instrumental learning. Learn. Mem..

[24] Zovkic I.B., Guzman-Karlsson M.C., Sweatt J.D. (2013). Epigenetic regulation of memory formation and maintenance. Learn. Mem..

[25] Mews P., Egervari G., Nativio R., Sidoli S., Donahue G., Lombroso S.I., Alexander D.C., Riesche S.L., Heller E.A., Nestler E.J. (2019). Alcohol metabolism contributes to brain histone acetylation. Nature.

[26] Subbanna S., Joshi V., Basavarajappa B.S. (2018). Activity-dependent Signaling and Epigenetic Abnormalities in Mice Exposed to Postnatal Ethanol. Neuroscience.

[27] García-Sevilla J.A., Ferrer-Alcón M., Martín M., Kieffer B.L., Maldonado R. (2004). Neurofilament proteins and cAMP pathway in brains of mu-, delta- or kappa-opioid receptor gene knock-out mice: Effects of chronic morphine administration. Neuropharmacology.

[28] Thomas G.M., Huganir R.L. (2004). MAPK cascade signalling and synaptic plasticity. Nat. Rev. Neurosci..

[29] Yang X., Wen Y., Zhang Y., Gao F., Yang J., Yang Z., Yan C. (2020). Dynamic Changes of Cytoskeleton-Related Proteins Within Reward-Related Brain Regions in Morphine-Associated Memory. Front. Neurosci..

[30] Valjent E., Pagès C., Hervé D., Girault J.-A., Caboche J. (2004). Addictive and non-addictive drugs induce distinct and specific patterns of ERK activation in mouse brain. Eur. J. Neurosci..

[31] Breen K.C., Robinson P.A., Wion D., Anderton B.H. (1988). Partial sequence of the rat heavy neurofilament polypeptide (NF-H) Identification of putative phosphorylation sites. FEBS Lett..

[32] Chwang W.B., Arthur J.S., Schumacher A., Sweatt J.D. (2007). The nuclear kinase mitogen- and stress-activated protein kinase 1 regulates hippocampal chromatin remodeling in memory formation. J. Neurosci..

[33] Zhang Y.X., Yang M., Liang F., Li S.Q., Yang J.S., Huo F.Q., Yan C.X. (2019). The pronociceptive role of 5-HT_6_ receptors in ventrolateral orbital cortex in a rat formalin test model. Neurochem. Int..

[34] Zhang Y., Yang J., Yang X., Wu Y., Liu J., Wang Y., Huo F., Yan C. (2020). The 5-HT_6_ Receptors in the Ventrolateral Orbital Cortex Attenuate Allodynia in a Rodent Model of Neuropathic Pain. Front. Neurosci..

[35] Hsu F.-S., Wu J.-T., Lin J.-Y., Yang S.-P., Kuo K.-L., Lin W.-C., Shi C.-S., Chow P.-M., Liao S.-M., Pan C.-I. (2019). Histone Deacetylase Inhibitor, Trichostatin A, Synergistically Enhances Paclitaxel-Induced Cytotoxicity in Urothelial Carcinoma Cells by Suppressing the ERK Pathway. Int. J. Mol. Sci..

[36] Lin W.-C., Hsu F.-S., Kuo K.-L., Liu S.-H., Shun C.-T., Shi C.-S., Chang H.-C., Tsai Y.-C., Lin M.-C., Wu J.-T. (2018). Trichostatin A, a histone deacetylase inhibitor, induces synergistic cytotoxicity with chemotherapy via suppression of Raf/MEK/ERK pathway in urothelial carcinoma. J. Mol. Med..

[37] Lu J.-C., Chang Y.-T., Wang C.-T., Lin Y.-C., Lin C.-K., Wu Z.-S. (2013). Trichostatin A modulates thiazolidinedione-mediated suppression of tumor necrosis factor α-induced lipolysis in 3T3-L1 adipocytes. PLoS ONE.

[38] Oride A., Kanasaki H., Mijiddorj T., Sukhbaatar U., Miyazaki K. (2014). Trichostatin A specifically stimulates gonadotropin FSHβ gene expression in gonadotroph LβT2 cells. Endocr. J..

[39] Yao J., Qian C.-J., Ye B., Zhang X., Liang Y. (2012). ERK inhibition enhances TSA-induced gastric cancer cell apoptosis via NF-κB-dependent and Notch-independent mechanism. Life Sci..

[40] Arany I., Herbert J., Herbert Z., Safirstein R.L. (2008). Restoration of CREB function ameliorates cisplatin cytotoxicity in renal tubular cells. Am. J. Physiol.-Renal Physiol..

[41] Sheng J., Lv Z., Wang L., Zhou Y., Hui B. (2011). Histone H3 phosphoacetylation is critical for heroin-induced place preference. Neuroreport.

[42] Paxinos G., Watson C. (1986). The Rat in Stereotaxic Coordinates.

[43] Zhao Y., Xing B., Dang Y.H., Qu C.L., Zhu F., Yan C.X. (2013). Microinjection of valproic acid into the ventrolateral orbital cortex enhances stress-related memory formation. PLoS ONE.

[44] Aida-Yasuoka K., Yoshioka W., Kawaguchi T., Ohsako S., Tohyama C. (2014). A mouse strain less responsive to dioxin-induced prostaglandin E2 synthesis is resistant to the onset of neonatal hydronephrosis. Toxicol. Sci..

[45] Hayashi H., Higashi T., Yokoyama N., Kaida T., Sakamoto K., Fukushima Y., Ishimoto T., Kuroki H., Nitta H., Hashimoto D. (2015). An Imbalance in TAZ and YAP Expression in Hepatocellular Carcinoma Confers Cancer Stem Cell-like Behaviors Contributing to Disease Progression. Cancer Res..

[46] Singh R., De Aguiar R.B., Naik S., Mani S., Ostadsharif K., Wencker D., Sotoudeh M., Malekzadeh R., Sherwin R.S., Mani A. (2013). LRP6 enhances glucose metabolism by promoting TCF7L2-dependent insulin receptor expression and IGF receptor stabilization in humans. Cell Metab..

[47] Salvucci O., Ohnuki H., Maric D., Hou X., Li X., Yoon S.O., Segarra M., Eberhart C.G., Acker-Palmer A., Tosato G. (2015). EphrinB2 controls vessel pruning through STAT1-JNK3 signalling. Nat. Commun..

[48] Farrell J.J., Sherva R.M., Chen Z.Y., Luo H.Y., Chu B.F., Ha S.Y., Li C.K., Lee A.C., Li R.C., Li C.K. (2011). A 3-bp deletion in the HBS1L-MYB intergenic region on chromosome 6q23 is associated with HbF expression. Blood.

